# The dilemma of routine testing for calcitonin thyroid nodule’s patients to detect or exclude medullary carcinoma: one single negative test should be valuable as rule-out strategy to avoid further calcitonin measurements over time

**DOI:** 10.1007/s12020-022-03047-2

**Published:** 2022-04-07

**Authors:** Pierpaolo Trimboli, Chiara Camponovo, Lorenzo Ruinelli

**Affiliations:** 1 Servizio di Endocrinologia e Diabetologia, Ospedale Regionale di Lugano, Ente Ospedaliero Cantonale (EOC), 6900 Lugano, Switzerland; 2 Facoltà di Scienze Biomediche, Università della Svizzera Italiana (USI), 6900 Lugano, Switzerland; 3 Team Data Science & Research, Area ICT, Ente Ospedaliero Cantonale, 6500 Bellinzona, Switzerland

**Keywords:** Calcitonin, Medullary thyroid carcinoma, Thyroid nodule, Routine testing

## Abstract

**Purpose:**

While calcitonin (CT) measurement is recognized as the most accurate tool to diagnose medullary thyroid carcinoma (MTC), its routine use in patients with thyroid nodule (TN) is not universally accepted. The present study raised the question whether a TN patient with an initial normal CT can have suspicious CT levels (i.e., at least >20 pg/ml) later during his follow-up.

**Methods:**

The historical database of our institution was searched to select TN patients undergone multiple CT tests, having an initial normal CT, and clinically followed up for years. The event of a CT above 20 pg/ml (mild-to-moderate suspicion) and 100 pg/ml (high suspicion) was searched in the follow-up of the included patients.

**Results:**

According to the study design, the study sample encompassed 170 patients (131 female, 39 male) with initial CT value ≤10 pg/ml. On the first CT test, patients were 54.8 years and median CT was 2.1 pg/ml in both females and males. Over a period of 14.5 years and a median clinical follow-up of patients of 53.0 (23.9–102.5) months, MTC could be excluded by histology or cytology in 109 (64%) and clinically in the remaining ones. On the follow-up over time, no patients had CT >20 pg/ml and only two cases had CT just above 10 pg/ml.

**Conclusion:**

According to the present results, one single CT testing with normal value could be reasonably used as a rule-out strategy in patients with TN to avoid further CT measurements.

## Introduction

Medullary thyroid carcinoma (MTC) is an uncommon malignancy originating from thyroid parafollicular C cells and familial in about one in four case [1]. Since C cells produce calcitonin (CT), the latter represents an excellent diagnostic marker to detect MTC in patients with thyroid nodule (TN). However, several technical factors can affect CT assay and other non-thyroidal pathologic conditions can increase CT [2, 3] thus limiting its routine use in clinical practice. In this context, a significant discrepancy between the major international societies exists. In 2006, European Thyroid Association (ETA) experts’ board recommended in favor of testing for CT all TN patients during their initial workup [4]. In 2009, the American Thyroid Association (ATA) position was neither for nor against the routine CT use [5]. The 2010 ETA guidelines joint with American Association of Clinical Endocrinologists (AACE) and Associazione Medici Endocrinologi (AME) introduced the concept to measure CT in subjects having family history of MTC/MEN, patients with indeterminate cytology or high-risk nodules at ultrasound, and in any patients undergoing thyroidectomy to avoid the risk of incomplete treatment when MTC is incidental [6]. In 2015, the ATA task force confirmed that they could not recommend for nor against the routine measurement of CT, although there was not uniform agreement on that [7].

Since the above issues, regardless of the potential aggressive behavior of MTC, whether or not testing for CT all TN patients represents an age-old dilemma [8–10]. In clinical practice, in the lack of solid evidence-based information about using CT and its specific cut-offs to indicate thyroidectomy, a CT value above 100 pg/ml is usually considered as suspicious/diagnostic for MTC and CT levels >20 pg/ml should be considered as mildly suspicious and may require further evaluations [2, 7, 11–13].

Starting from all the above issues, the present study was undertaken to evaluate what is the meaning of a single normal CT value in the history of TN patients. To the best of our knowledge, no study systematically investigated the CT trend in TN patients with initial normal CT. Then, the aim of the present study was to evaluate whether a TN patient with an initial normal CT value can have suspicious CT levels (i.e., at least >20 pg/ml) during his follow-up. Accordingly, the trend of CT levels over time in TN patients with initial normal CT and followed up at our institution was analyzed, and their clinical data reviewed. Specifically, the study outcome measures were the following: (a) subsequent CT evaluations over time, and (b) histology and cytology, when available.

## Methods

### Study design and patients’ selection

Based on the study conceptualization, we aimed to find a specific setting of cases, such as TN patients having at least one CT test with negative result and at least a second CT test over time, and undergone a thyroid visit. The study period was set from January 2007 to April 2021. Accordingly, the institutional database of all patients undergone CT measurement during the study period was screened. As the essential inclusion criterion, only those patients undergone also thyroid examination in the thyroid diseases-dedicated services of our institution could be enrolled. After recording all cases tested for CT, they were excluded patients: (1) undergone only one single CT testing, (2) undergone CT testing after previous thyroidectomy or any other MTC-related treatment, (3) with data of CT measurement in non-serum sample (i.e., washout fluids from biopsy or fine-needle aspiration), (4) with incomplete/unavailable clinical data, and (5) refusing to be included in this study.

### Reference standard

As the standard of reference against to which the baseline negative CT was tested, we considered CT ranges generally recognized as associated with different suspicion for MTC: >10 and <20 pg/ml (just above the upper normal reference), >20 and ≤100 pg/ml (mild-to-moderate suspicion), and >100 pg/ml (high suspicion) [2, 7, 11–13]. The histological diagnosis was the gold standard for diagnosis. In the absence of histology, the cytological report after fine-needle aspiration (FNAC) and the last clinical diagnosis was used as reference.

### Laboratory tests

CT was measured on IMMULITE^®^ 2000 XPi platform (Siemens Healthcare Diagnostics) until February 2019 and on Cobas 8000 platform (Roche Diagnostics) later, according to the manufacturer instructions.

### Statistical analysis

All continuous variables were analyzed by nonparametric tests and expressed through the manuscript as median and interquartile ranges (IQR). Comparison of paired and unpaired data from two groups were compared by Mann–Whitney test. The negative predictive value (NPV) of initial negative CT to exclude subsequent clinically significant increase of CT was estimated against the multiple repeated CT tests with value above 10 or 20 pg/ml. The NPV of initial negative CT to exclude MTC was calculated using histology and cytology, when available. The correlation between thyroid volume (calculated by using the ellipsoid volume formula applied for each thyroid lobe and expressed in ml) and CT value was analyzed by linear regression.

## Results

### Flow of patients and data

After the initial screening of the institutional database a number of 1935 patients undergone both CT testing and thyroid visit were found. Among these, according to the study selection criteria, 1657 cases were excluded. The initial series of 278 patients with at least two CT measurements was reviewed and 108 cases were excluded because of basal CT higher than 10 pg/ml (i.e., CT value above 100 pg/ml [*n* = 8 cases], or CT between 10 and 100 pg/ml [*n* = 23 cases of whom one with preoperative CT of 16.41 pg/ml and histological diagnosis of diffuse C cell hyperplasia) or other reasons (CT measurement during postoperative follow-up of MTC, non-nodular disease, unavailable data, refusal to be included). Finally, the study series encompassed 170 patients having initial CT ≤10 pg/ml of whom 115 with multiple nodules.

### Baseline features of the study series

The study series encompassed 170 patients (131 female, 39 male) with initial CT value ≤10 pg/ml of whom 136 with undetectable level. On the first CT test, patients’ median age was 54.8 (43.4–65.4) years, while median CT value was 2.1 (2.1–2.1) pg/ml. The median CT value recorded in both females and males was 2.1 (2.1–2.1) pg/ml.

### Clinical follow-up

The whole study period was 14.5 years. The median time of clinical follow-up of patients (i.e., between first and last clinical evaluation) was 53.0 (23.9–102.5) months. During the follow-up, 50 patients underwent thyroid surgery (19 papillary thyroid carcinomas, 2 noninvasive follicular thyroid neoplasm with papillary-like nuclear features [NIFTP], and 29 benign goiters), 59 were submitted to biopsy, the remaining 61 were clinically observed, and no patient was diagnosed with MTC.

### Analysis of repeated calcitonin

**Fig. 1:**
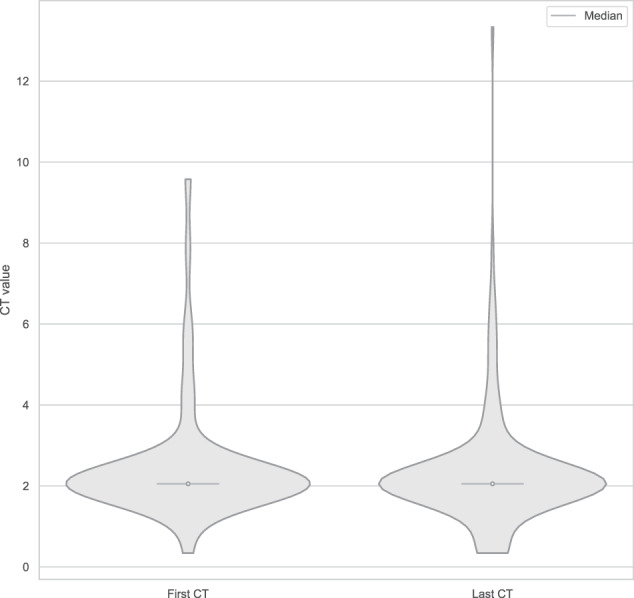
Distribution of CT values at first and last test. The wideness of violin indicates the density of cases with the same CT value. The height of violin indicates the ranges of CT values

The median age on the last CT test was 56.8 (46.0–68.3) years. Figure 1 reports the violin plot of CT values recorded at first and last CT test.

Among the 170 patients of the study series, 120 underwent CT tests two times, 28 three times, and the remaining 22 more than three times. On the last CT test, 124 patients had undetectable value.

**Fig. 2:**
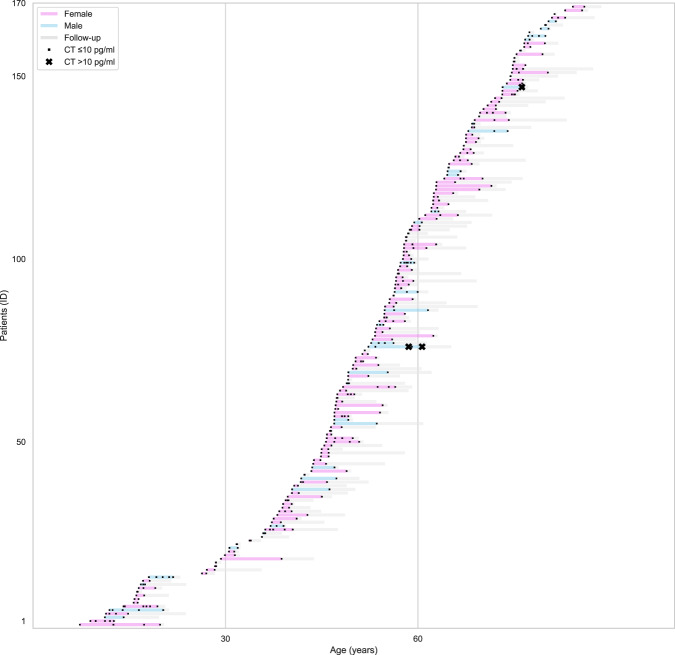
CT results in all patients of the study series according to their age on the time of each test. In *y* axis the Id of any patient and in *x* axis her/his age in years. Each patient is represented by a horizontal line with color according to her/his gender. Dot indicates CT tests with value ≤10 pg/ml. X represents CT test >10 pg/ml. Gray line represents the overall period of clinical follow-up

Figure 2 illustrates all data of CT and clinical follow-up of all patients according to their age on the time of CT tests. Median interval between last CT test and last clinical visit was 17.3 (8.1–63.5) months. During the CT follow-up over time, no patients had CT >20 pg/ml. There were two patients with CT value above 10 pg/ml over time. The first one underwent three CT tests over 3.3 years (2.05, 10.26, and 2.05 pg/ml); the second one underwent five CT tests over a period of 8 years (9.57, 8.89, 10.26, 7.18, and 13.3 pg/ml).

### Predictivity tests

The NPV of an initial negative CT to not face over time a CT above 10 and 20 pg/ml was 98.8 and 100%, respectively. When we consider only the subgroup of those patients who underwent surgery or FNAC, NPV of initial normal CT to exclude MTC was 100%. No significant difference was found between the two CT assays used in our institutional series in terms of NPV.

### Evaluation of determinants and potential interfering factors of CT values

As above reported, no significant difference in CT was found between males and females.

TSH value was available in 167/170 patients with median value 1.1 (0.7–1.9) mIU/l. Among these, TSH was higher than upper reference in 11 (6.5%) and lower than the lower reference in 9 (5.4%) cases. No significantly different CT value was found among these subgroups.

There was a subgroup of 116 patients tested for anti-thyroid antibodies of which 75 with positive values (i.e., autoimmune thyroiditis). When we compare CT values between positives and negatives subgroups, no significant difference was found in both first and last CT test.

Data about ultrasound estimated thyroid volume were available in 84/170 patients with median value of 18 (12.5–30) ml. No positive correlation between CT value and thyroid size was observed.

Unfortunately, data about smoking was not extractable in our database.

## Discussion

Diagnosing MTC is still a challenge [2]. FNAC performance on this cancer is much lower than that generally estimated for thyroid malignancy [14, 15] while serum CT testing is highly reliable to detect MTC [16]. However, since MTC is a rare tumor, as its clinical and ultrasound presentation is heterogeneous, and because several factors can interfere on the CT measurement with the possibility to have false-positive value with consequent difficult interpretation, the routinely testing for CT in all the huge mass of subjects with TNs has not been accepted [6, 7]. In this highly complicated field, the present study raised the question whether using a single CT measurement as rule-out strategy can be an adequate compromise.

The whole database from 2007 to 2021 of our institution was screened and a number of 170 TN patients undergone at least two CT testing was found. As the major result from our study was that one single CT testing in TN patients can be used as rule-out strategy to exclude MTC. In fact, when CT is negative (i.e., <10 pg/ml) once, the likelihood to have mildly suspicious CT (i.e., >20 pg/ml) later, over the time, is zero. Importantly, the largest part of patients were clinical followed-up for long-term follow-up as demonstrated by the fact that the upper IQR range of clinical follow-up period between the last CT test and the last clinical visit was larger than 5 years while that between the first CT test and the last clinical visit was 8 years and half. In addition, MTC was excluded by FNAC or histology in 109/170 (64%) patients of the series. All in all, only two patients had CT slightly above than 10 pg/ml. These values might be due to some interfering factors or other non-thyroidal pathologic conditions [2, 3]. Anyway, both patients were males and a higher CT can be expected than females.

Potential determinants and interfering factors of CT measurement were analyzed. No significant difference was found between males and females. In agreement with Grani et al. [17] no significant difference was found between patients with or without anti-thyroid antibodies. No positive correlation between CT value and thyroid size was observed, differently from other studies enrolling healthy patients [18]. All in all, these findings could indirectly suggest that the threshold of 10 pg/ml can overcome the potential interferences from these parameters and features.

To collect our final study series, we initially searched in our database for all cases undergone CT, and we initially found 1935 patients of whom the largest part was excluded according to both study selection criteria and aim. Even if this study focused only on those patients with normal CT, a brief discussion about the prevalence of MTC might be addressed. Here, 11 MTCs were found with an overall prevalence of 0.56%. Although the present is not a consecutive series undergoing CT testing, this percentage appears not dissimilar to that recorded by Elisei et al. [10], such as 0.40%, in a large sample of 10,864 patients undergone routine CT evaluation in the 90’s. In this context, Costante et al. [9] found 15 MTC and seven C cell hyperplasia (0.37%) in 5817 consecutive patients. Furthermore, a recent meta-analysis included 17 trials and a total 74,407 patients with nodular thyroid disease, the prevalence of MTC was found between 0.11 and 0.85% [19]. Present data corroborate that MTC is not an infrequent finding among patients with TNs.

Having normal CT is not 100% proof of the absence of MTC of course. However, that possibility remains quite rare. The most relevant study in this context was published by Chambon et al. [20]. There, a series of 2733 patients undergoing thyroidectomy for nodular goiter were submitted to preoperative CT, 45 (1.64%) MTCs were found at histology, and two millimetric MTCs (sized 1 and 4 mm, respectively) had a presurgical normal CT levels. From a prognostic point of view, it has to be underlined that very small MTCs are usually different from large ones, identifying microMTC is challenging and clinically significant only in hereditary MTCs, and in any case the incidence of distant metastases in microMTCs was reported as only 1.3% [21]. Anyway, normal CT levels do not require specific clinical actions. Beside all this, the possibility of non-secreting MTC exists and this very rare scenario should be always taken into account [22].

The present study raises the old-age question whether using or not the routine testing for CT in patients with nodular goiter. Since a 10-year survival of MTC patients was reported as 50%, the major chance we have to improve their cure and survival is to achieve their early diagnosis and surgical treatment [23]. One of the main aims of routinely measuring CT is to detect early MTCs [3, 24, 25]. The present data should encourage to consider that one single CT with normal value as a reliable proof of the absence of MTC in a TN patients. In fact, the largest part of patients with nodular goiter neither has increase of nodule(s) volume nor, importantly, develops new nodules over time [26]. Then, only a very small proportion of TN patients should undergo a repeated CT testing in case of TNs change. From a cost-effectiveness point of view, testing for CT all TN patients could be adopted in clinical practice to avoid further costs of repeating CT test [27]. On the other hand, this is a strategy quite similar to that of performing FNAC in nodules with no strict indication for cytological assessment (i.e., low- to intermediate-risk lesions with size higher than 2–1.5 cm). In this setting, achieving a solid proof of benignancy means generally to address patients to safe clinical follow-up only.

Some limitations of the present study should be disclosed. The present series was not consecutive. All data were retrospectively extracted. The patients included in the present study were managed by several physicians. Institutional guidelines about using CT have not been implemented; then, the indication to repeat CT testing could not be extracted. Since the present was not a consecutive series, a true cost analysis could not be attempted. Two kits for CT were used in out institution during the study period. As strengths of this study, it is the first analysis of repeated CT after an initial normal CT value, a long-term clinical follow-up of patients was available, all patients were managed by services of our institutions dedicated to thyroid diseases, two thirds of cases was submitted to FNAC or histology.

In conclusion, the present study showed that patients with TNs having an initial normal CT have no risk to develop suspicious CT values (i.e., >20 pg/ml) during their subsequent clinical follow-up. Rarely, a slight increase of CT (i.e., >10 pg/ml) occurs with no clinical significance. Thus, one single CT testing with normal value could be reasonably used as a rule-out strategy in patients with nodular goiter to avoid further CT measurements over time.

## Data Availability

The data sets used and/or analyzed during the current study are available from the corresponding author on reasonable request.

## References

[1] Tuttle RM, Ball DW, Byrd D (2010). Medullary carcinoma. J. Natl Compr. Canc Netw..

[2] Trimboli P, Giovanella L, Crescenzi A, Romanelli F, Valabrega S, Spriano G, Cremonini N, Guglielmi R, Papini E (2014). Medullary thyroid cancer diagnosis: an appraisal. Head. Neck..

[3] Elisei R, Romei C (2013). Calcitonin estimation in patients with nodular goiter and its significance for early detection of MTC: european comments to the guidelines of the American Thyroid Association. Thyroid Res..

[4] Schlumberger M, Bastholt L, Dralle H, Jarzab B, Pacini F, Smit JWA (2012). European Thyroid Association guidelines for metastatic medullary thyroid cancer. Eur. Thyroid J..

[5] Kloos RT, Eng C, Evans DB, Francis GL, Gagel RF, Gharib H, Moley JF, Pacini F, Ringel MD, Schlumberger M (2009). Medullary thyroid cancer: management guidelines of the American Thyroid Association. Thyroid.

[6] Gharib H, Papini E, Paschke R (2010). American Association of Clinical Endocrinologists, Associazione Medici Endocrinologi, and European Thyroid Association medical guidelines for clinical practice for the diagnosis and management of thyroid nodules: executive summary of recommendations. J. Endocrinol. Invest.

[7] Haugen BR, Alexander EK, Bible KC, Doherty GM, Mandel SJ, Nikiforov YE, Pacini F, Randolph GW, Sawka AM, Schlumberger M, Schuff KG, Sherman SI, Sosa JA, Steward DL, Tuttle RM, Wartofsky L (2016). 2015 American Thyroid Association Management Guidelines for Adult Patients with Thyroid Nodules and Differentiated Thyroid Cancer: The American Thyroid Association Guidelines Task Force on Thyroid Nodules and Differentiated Thyroid Cancer. Thyroid.

[8] Rieu M, Lame MC, Richard A, Lissak B, Sambort B, Vuong-Ngoc P, Berrod JL, Fombeur JP (1995). Prevalence of sporadic medullary thyroid carcinoma: the importance of routine measurement of serum calcitonin in the diagnostic evaluation of thyroid nodules. Clin. Endocrinol..

[9] Costante G, Meringolo D, Durante C, Bianchi D, Nocera M, Tumino S, Crocetti U, Attard M, Maranghi M, Torlontano M (2007). Predictive value of serum calcitonin levels for preoperative diagnosis of medullary thyroid carcinoma in a cohort of 5817 consecutive patients with thyroid nodules. J. Clin. Endocrinol. Metab..

[10] Elisei R, Bottici V, Luchetti F, Di Coscio G, Romei C, Grasso L, Miccoli P, Iacconi P, Basolo F, Pinchera A, Pacini F (2004). Impact of routine measurement of serum calcitonin on the diagnosis and outcome of medullary thyroid cancer: experience in 10,864 patients with nodular thyroid disorders. J. Clin. Endocrinol. Metab..

[11] Machens A, Hoffmann F, Sekulla C, Dralle H (2009). Importance of gender-specific calcitonin thresholds in screening for occult sporadic medullary thyroid cancer. Endocr. Relat. Cancer.

[12] Ahmed SR, Ball DW (2011). Clinical review: Incidentally discovered medullary thyroid cancer: diagnostic strategies and treatment. J. Clin. Endocrinol. Metab..

[13] Dora JM, Canalli MH, Capp C, Puñales MK, Vieira JG, Maia AL (2008). Normal perioperative serum calcitonin levels in patients with advanced medullary thyroid carcinoma: case report and review of the literature. Thyroid.

[14] Trimboli P, Treglia G, Guidobaldi L, Romanelli F, Nigri G, Valabrega S, Sadeghi R, Crescenzi A, Faquin WC, Bongiovanni M, Giovanella L (2015). Detection rate of FNA cytology in medullary thyroid carcinoma: a meta-analysis. Clin. Endocrinol. (Oxf.)..

[15] Trimboli P, Giannelli J, Marques B, Piccardo A, Crescenzi A, Deandrea M (2022). Head-to-head comparison of FNA cytology vs. calcitonin measurement in FNA washout fluids (FNA-CT) to diagnose medullary thyroid carcinoma. A systematic review and meta-analysis. Endocrine.

[16] Bugalho MJ, Santos JR, Sobrinho L (2005). Preoperative diagnosis of medullary thyroid carcinoma: fine needle aspiration cytology as compared with serum calcitonin measurement. J. Surg. Oncol..

[17] Grani G, Nesca A, Del Sordo M, Calvanese A, Carbotta G, Bianchini M, Fumarola A (2012). Interpretation of serum calcitonin in patients with chronic autoimmune thyroiditis. Endocr. Relat. Cancer.

[18] Giovanella L, Imperiali M, Ferrari A, Palumbo A, Lippa L, Peretti A, Graziani MS, Castello R, Verburg FA (2012). Thyroid volume influences serum calcitonin levels in a thyroid-healthy population: results of a 3-assay, 519 subjects study. Clin. Chem. Lab. Med..

[19] Vardarli I, Weber M, Weidemann F, Führer D, Herrmann K, Görges R (2021). Diagnostic accuracy of routine calcitonin measurement for the detection of medullary thyroid carcinoma in the management of patients with nodular thyroid disease: a meta-analysis. Endocr. Connect..

[20] Chambon G, Alovisetti C, Idoux-Louche C, Reynaud C, Rodier M, Guedj AM, Chapuis H, Lallemant JG, Lallemant B (2011). The use of preoperative routine measurement of basal serum thyrocalcitonin in candidates for thyroidectomy due to nodular thyroid disorders: results from 2733 consecutive patients. J. Clin. Endocrinol. Metab..

[21] Machens A, Dralle H (2012). Biological relevance of medullary thyroid microcarcinoma. J. Clin. Endocrinol. Metab..

[22] Trimboli P, Giovanella L (2015). Serum calcitonin negative medullary thyroid carcinoma: a systematic review of the literature. Clin. Chem. Lab. Med..

[23] Kebebew E, Ituarte PH, Siperstein AE (2000). Medullary thyroid carcinoma: clinical characteristics, treatment, prognostic factors, and a comparison of staging systems. Cancer.

[24] Valle LA, Kloos RT (2011). The prevalence of occult medullary thyroid carcinoma at autopsy. J. Clin. Endocrinol. Metab..

[25] Pillarisetty VG, Katz SC, Ghossein RA, Tuttle RM, Shaha AR (2009). Micromedullary thyroid cancer: how micro is truly micro?. Ann. Surg. Oncol..

[26] Durante C, Costante G, Lucisano G, Bruno R, Meringolo D, Paciaroni A, Puxeddu E, Torlontano M, Tumino S, Attard M, Lamartina L, Nicolucci A, Filetti S (2015). The natural history of benign thyroid nodules. JAMA.

[27] Cheung K, Roman SA, Wang TS, Walker HD, Sosa JA (2008). Calcitonin measurement in the evaluation of thyroid nodules in the United States: a cost-effectiveness and decision analysis. J. Clin. Endocrinol. Metab..

